# Effects of Plasmonic Metal Core -Dielectric Shell Nanoparticles on the Broadband Light Absorption Enhancement in Thin Film Solar Cells

**DOI:** 10.1038/s41598-017-08077-9

**Published:** 2017-08-09

**Authors:** Peng Yu, Yisen Yao, Jiang Wu, Xiaobin Niu, Andrey L. Rogach, Zhiming Wang

**Affiliations:** 1 Institute of Fundamental and Frontier Science, University of Electronic Science and Technology of China, Chengdu, 610054 P. R. China; 2 Department of Electronic and Electrical Engineering, University College London, Torrington Place, London, WC1E 7JE United Kingdom; 3 State Key Laboratory of Electronic Thin Film and Integrated Devices, University of Electronic Science and Technology of China, Chengdu, 610054 P.R. China; 4 Department of Physics and Materials Science and Centre for Functional Photonics (CFP), City University of Hong Kong, Hong Kong SAR, P. R. China

## Abstract

To guide the design of plasmonic solar cells, theoretical investigation of core (metal)-shell (dielectric) nanoparticles for light absorption enhancement in thin film Si solar cells is performed. In contrast to the reported simulations and experimental results that rear-located surface plasmon on bare metallic nanoparticles is preferred, the core-shell nanoparticles demonstrate better performance when surface plasmon is located in front of a solar cell. This has been attributed to the enhanced forward scattering with vanishing backward scattering preserved over a wide spectral range in core-shell nanoparticles. This work provides a concept to achieve enhanced forward scattering with weakened backward scattering in plasmonic thin film solar cells.

## Introduction

Solar energy is an ideal candidate for addressing the energy crisis and reducing carbon emission. Efficient light management schemes are crucially important to maintain high efficiency of photovoltaic (PV) devices such as thin film solar cells, quantum dot solar cells, nanostructured solar cells, and plasmonic solar cells. Thin film solar cells offer the advantage of reduced material costs, but they incur transmission losses and reduce cell performance as absorber layers become thinner^1^. Surface texturing is the main method for commercial solar cells to boost efficiencies, but it is not compati﻿ble with thin film solar cells﻿. Quantum dot (QD) solar cells have the potential to boost the maximum attainable thermodynamic conversion efficiency of solar photon conversion up to ~66%^2^. However, they are still suffering from poor optical and electrical properties of QDs grown by mainstream Stranski-Krastanov method, because the strain accumulation from the stacking multiple QDs introduces lattice defects which are detrimental to device performance^3^. The nanostructured solar cells such as those based on the close-packed nanowires^4–6^ are beneficial for the absorption at longer wavelengths, but the device performance is vulnerable to the surface recombination due to the large surface-area-to-volume ratio^7^.

The plasmonic light-trapping scheme attracted a lot of attention to aid photocurrent enhancement in solar-cells^8–13^. Incorporating plasmonic enhancers into thin film solar cells helps to achieve higher power conversion efficiency (PCE) while lessening physical thickness of the materials significantly. This approach makes use of collective oscillation of excited free electrons in noble metal nanoparticles, which are influenced by particle shape, size and dielectric properties of the surrounding medium, and surface coverag^14^. Catchpole et al. have shown that metal nanoparticles with cylindrical and hemispherical geometry lead to a much higher path length enhancements than their spherical counterpart^15^. Another of their works demonstrated that resonant surface plasmon polariton modes, supported by disk-shaped metal nanoparticles on high-index substrates, is very sensitive to the area in contact with the substrate and insensitive to particle height^16^. J. Wu et al. coupled Au and Ag nanoparticles on InAs/GaAs QD solar cell surfaces and presented an overall PCE enhancement from 8.0% to 9.5% and 8.9% respectively^17^. Size-controlled Ag nanoparticles with high aspect-ratio resulted in an 8% increase in the short circuit density of GaAs solar cells^18^. Self-assembled Ag nanoparticle-based plasmonic back reflector in a-Si:H cell achieved a short circuit current as high as 15.1 mA/cm^2^ ^19^. Metal nanostructured plasmonic sources, such as nanospheres^17–19^, nanostars^20^, nanocages^21^, nanodisks^22^, nanocavity^23^, nanovoids^24^, nucleated nanoparticles^25^, have been all demonstrated for optical absorption enhancement. Plasmonics also opens an avenue for achieving panchromatic solar cell conversion designs, especially for quantum dot solar cells. Our previous work provides a proof-of-concept demonstration for addressing the weak absorption of QDs by utilizing multibranched Au nanostars^20^.

However, bare metal nanoparticles, serving as recombination centers when coupled into solar cells, are suffering from energy losses. Their exposure to air and moisture may give rise to modification of the scattering pattern^26^. Certain metals can give rise to a diffusion of the metal contamination into the active layer of solar cells, which will have deleterious effects. For that reason, core-shell nanostructures composed of plasmonic metals and dielectric (or semiconductor) shells are receiving extensive attention^27^. These hybrid nanostructures not only ensure electrical and chemical isolation of the plasmonic core, but also provide another possibility to tune the localized surface plasmon resonance (LSPR) because it is dependent on the dielectric function of the surrounding medium^28, 29^. The combination of Au and temperature-triggered VO_2_ showed switchable optical properties^30^. Metal/semiconductor nanoparticles yield plasmon resonance properties that are distinct from those of their bare metal counterparts. Fano resonance was observed in (Au core) / (ZnS shell) and (Au core) / (CdS shell) structures^31^. To design scattering objects insensitive to angle incidence (a key consideration in plasmonic solar cell design), a method, with a combination of high permittivity shell and a metal core, was proposed for achieving unidirectional scattering^32^. Few papers have reported optical enhancement via coupling the core-shell structures into solar cells^33, 34^. The major aim of this paper is to shed light on the difference in performance of core-shell architectures deposited on top/back of a thin film Si solar cell.

## Results

### Optical properties of core-shell nanoparticles

**Figure 1:**
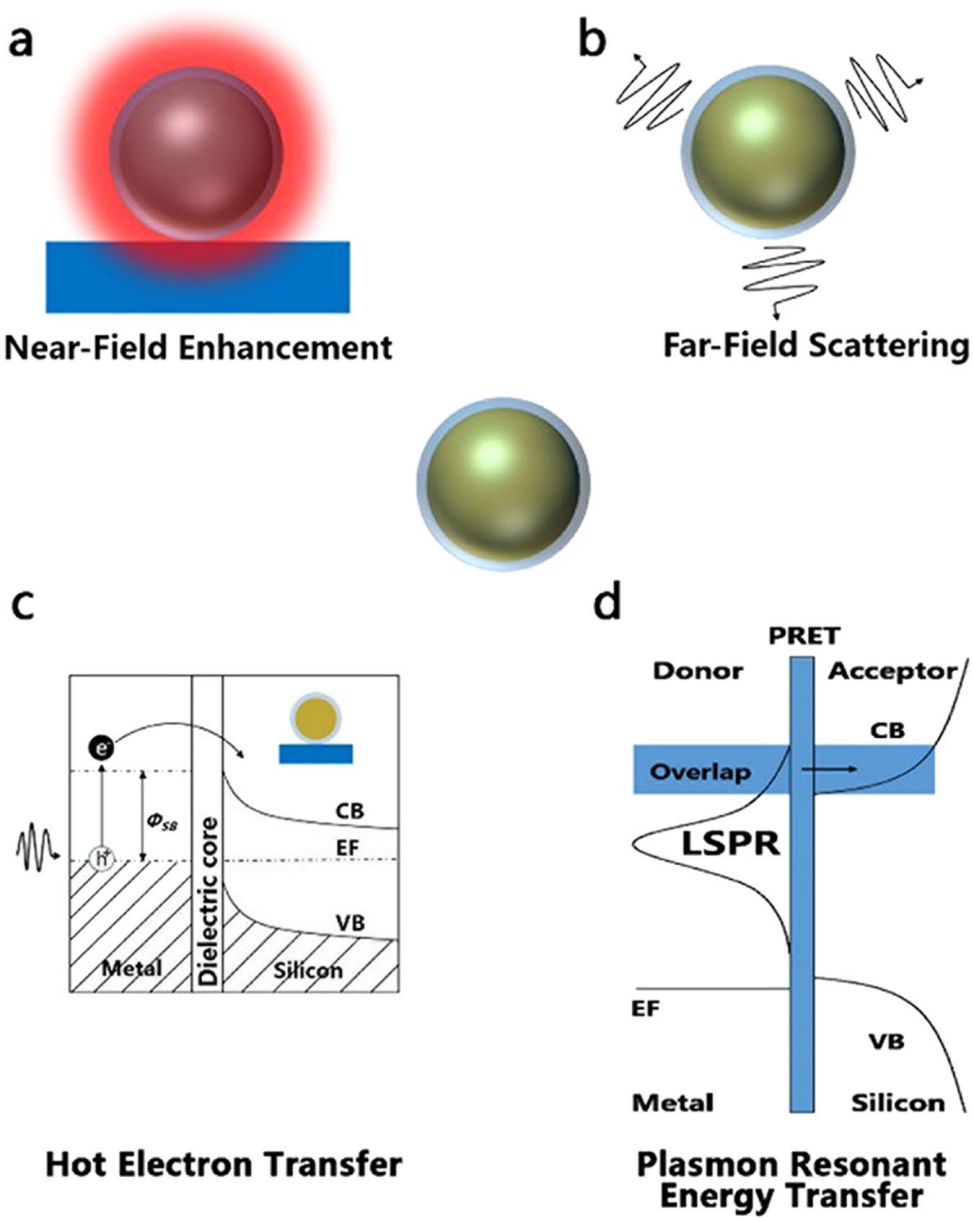
Four possible photophysical processes of core-shell nanoparticle in contact with Si solar cells. (**a**) Near-field coupling. (**b**) Far-field scattering. (**c**) Hot electron transfer. (**d**) Plasmon resonant energy transfer.

The fundamental underpinnings of core-shell plasmon enhanced phenomena for solar cell applications depend on four mechanisms, namely near-field coupling, far-field scattering, hot electron transfer (HET) and plasmon resonant energy transfer (PRET), as shown in Fig. 1. Near-field enhancement and far-field scattering are radiative effects. Electromagnetic near-field enhancement is the consequence of the interaction of plasmonic nanostructures with incident light, as illustrated in Fig. 1a. In the vicinity of the nanostructure, the intensities of these fields are typically orders of magnitude higher than the incident light. The near-fields at the plasmon resonance decay proportional to r^−6^, where r is the distance from the nanostructure, typically <50 nm from the nanostructure surface. Thus, enhanced fields generate more electron-hole pairs. It is worth noting that the scattered photon energy remains unchanged so that only the light within the band gap is utilized. Figure 1b depicts the far-field scattering mode. Incident light on metal nanoparticles with a sufficiently high albedo is scattered into the far-field within distances of several hundred nanometres and finally leads to multiple scattering. The scattered light is absorbed by under-layered bulk materials. For concentric core-shell nanostructures, they have the ability to polarize the incoming light^35^. Per Mie theory, far-field effects can be tailored as a function of the surrounding permittivity around nanostructures. The dielectric coatings with high refractive index are able to shift the LSPR frequencies to red region enabling capture of solar photons where transmission losses are most significant for thin film Si solar cells. In addition to enhancing near-infrared absorption, they enable preferential scattering in targeted directions^36^. The HET and PRET are both non-radiative effects. As demonstrated in Fig. 1c, hot electrons with sufficient energy can overcome the Schottky barrier, *Φ*_*SB*_, and finally be injected into the semiconductor conduction band. However, the coating layer must be thinner than the electron tunnelling barrier^37^. In PRET process, a plasmonic metal absorbs sunlight, then transfers the absorbed energy from the metal to a semiconductor via dipole–dipole coupling, which generates electron–hole pairs below and near the semiconductor band edge^38^. In this paper, optical near-field enhancement and far-field scattering of core-shell particle are discussed.

**Figure 2:**
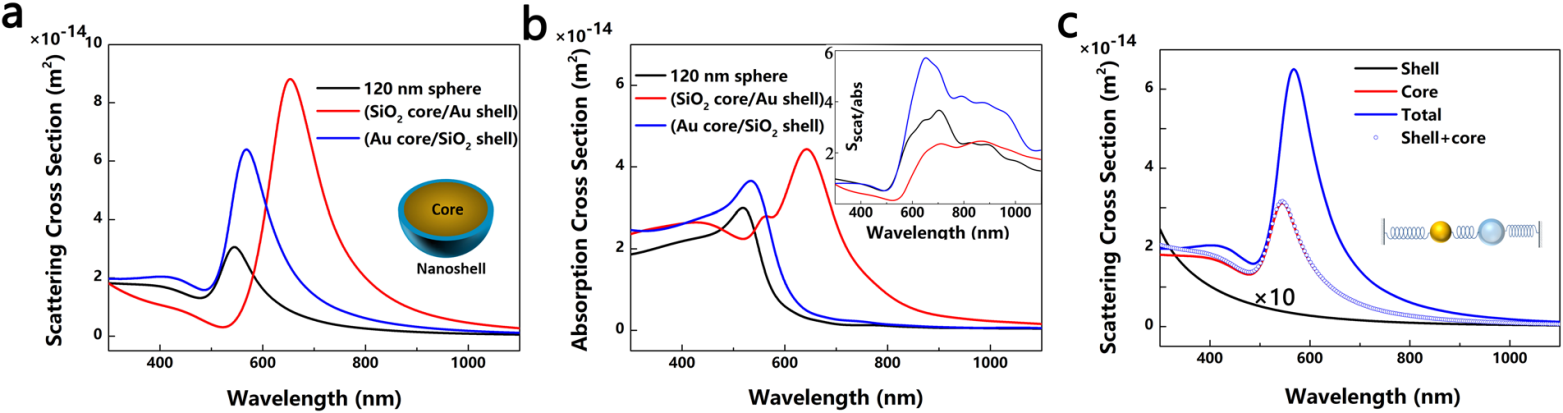
Scattering (**a**) and absorption (**b**) spectra of the single core-shell nanosphere calculated per Mie theory. A medium of index 1 was used in background. The inset in (**b**) is the scattering-to-absorption ratio ***S***, indicating that the core-shell nanostructure has broadband external field enhancement which exceeds parasitic absorption. The diameter of the core is set as 120 nm and the thickness of the coating shell ***t*** is 15 nm. (**c**) The individual scattering spectra of an Au core, a hollow SiO_2_ shell, a core-shell nanosphere and the sum value of an Au core and a hollow SiO_2_ shell. The inset schematically illustrates the oscillator model. The golden sphere and the blue shell stand for the Au core and the SiO_2_ shell, respectively.

The introduction of the coating shell increases the light scattering, leading to a higher scattering peak than that of the unshelled bare Au nanosphere, as illustrated in Fig. 2, with consideration of both near- and far-field effects. The asymmetric line shape of Fano resonance on scattering cross section in Fig. 2a is observed in this hybrid nanostructure. Although the most efficient method for inducing Fano resonance in plasmonic metal nanoparticle is the introduction of the symmetry breaking, it was also shown for the core-shell nanostructures without symmetry breaking in recent simulation and experimental studies^31, 39^. Since there is no symmetry breaking in our simulation, the signature of Fano resonance is ascribed to the coupling interaction between the metal core and the dielectric shell. The free electron oscillation excited in an Au core and the polarization charge oscillation in a shell under optical electric field interact with each other through Coulombic interaction. Moreover, the dielectric shell can transfer energy to free electrons in the Au core, giving rise to enhanced plasmon resonance. While the scattering intensity of SiO_2_ core/Au shell is larger than that of the Au core/SiO_2_ shell in the near infrared region, the scattering-to-absorption ratio (***S*** = σ_scat_/σ_abs_) of the Au core/SiO_2_ shell is predominant over 300–1100 nm, as shown in Fig. 2b, indicating the core/metal-shell/dielectric structure is favored in solar cell application. The Fano resonance of the core-shell nanoparticle can be analyzed using an intuitive mechanical model that constitutes two oscillators^40^, as the inset demonstrated in Fig. 2c. Figure 2c plots the scattering spectra of an individual Au core, a hollow SiO_2_ shell, a core-shell nanosphere, and the sum value of Au core and hollow SiO_2_ shell. The hollow shell with the absence of Au core demonstrates an increasing scattering intensity in the short-wavelength region. When the core and the shell are combined, the structures shows strengthened scattering peak with a clear asymmetric Fano resonance compared with that of the sum value of individual components due to the constructive interference of two oscillators.

**Figure 3:**
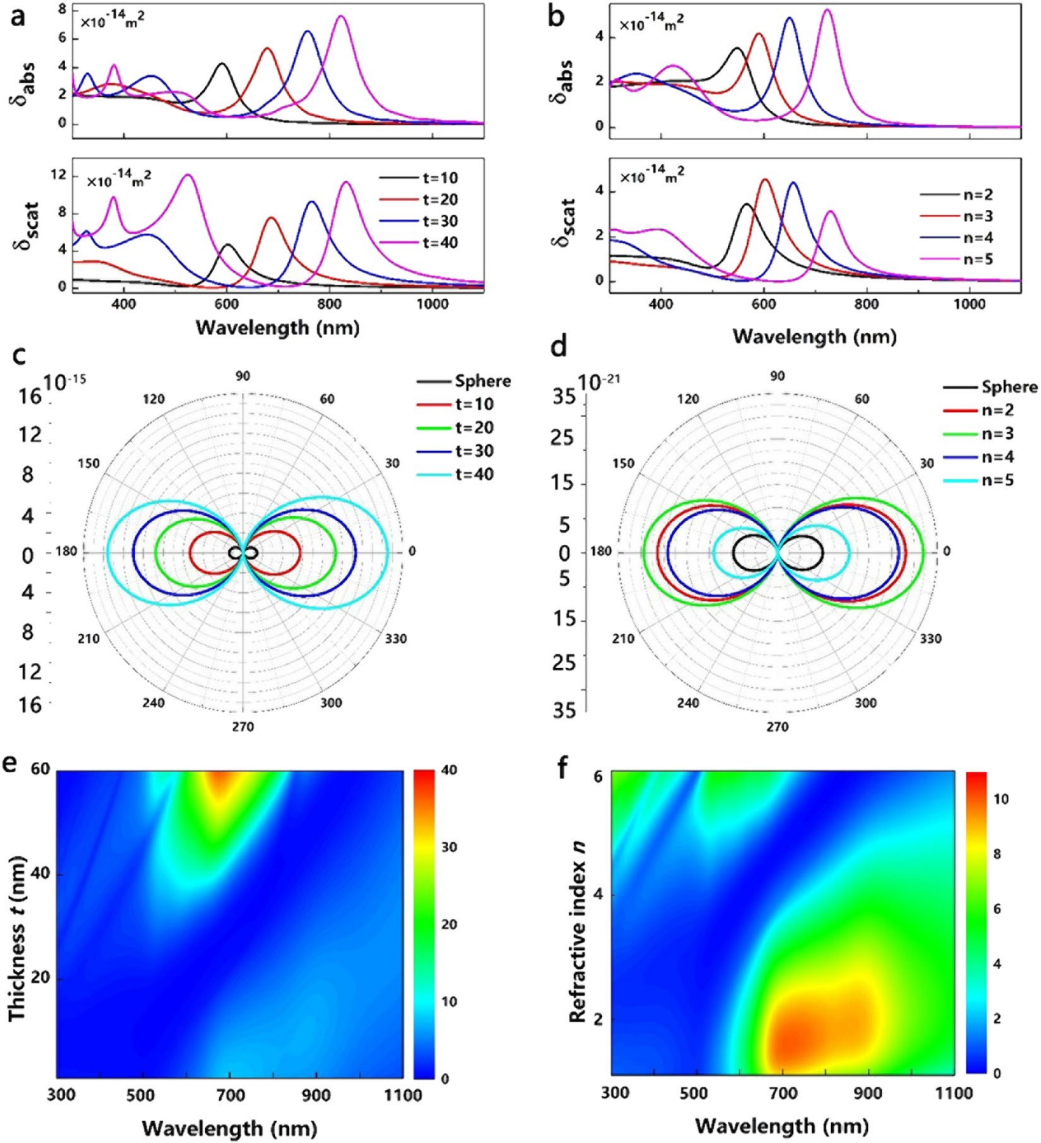
Scattering cross section and absorption cross section of a 100 nm Au nanosphere with (**a**) Coating thickness ***t*** = 10, 20, 30, 40 nm. (**b**) With refractive index of coating dielectric ***n*** = 2, 3, 4, 5. Angular scattering (a. u.) of (**c**) a 100 nm Au nanosphere with coating thickness ***t*** = 10, 20, 30, 40 nm. The refractive index of coating dielectric index ***n*** is set as 3. (**d**) 100 nm Au nanosphere with ***n*** = 2, 3, 4, 5; ***t*** = 10 nm. The Y-axis value is square of the scattering intensity. Calculated scattering-to-absorption cross section (***S***) as (**e**) the function of ***t*** with ***n*** = 3.4 and a 120 nm Au core. (**f**) As the function of ***n*** with ***t*** = 15 nm and a 120 nm Au core.

Figure 3 plots σ_scat_, σ_abs_ and square of angular scattering intensities of the core-shell nanoparticles with different coating refractive index ***n*** and thickness ***t***. As the coating thickness increases, the σ_scat_ and σ_abs_ increase because they are dependent on nanoparticle volume or size per Mie theory (scales with *a* ^6^ for σ_scat_, where *a* is nanoparticle size), as shown in Fig. 3a. However, as ***n*** increases, the scattering intensity maximizes at their resonant wavelengths when ***n*** = ~3 and the parasitic absorption increases, as illustrated in Fig. 3b, which contradicts results with Ag nanoparticle embedded in homogeneous dielectric matrix^41^. This phenomenon can be attributed to the difference of complex dielectric functions of materials under dipole approximation. Multiple peaks, especially under large ***t*** and ***n***, are evident in Fig. 3a,b due to excitation of higher order resonances appears (e.g. quadruple or octupole moments). The far-field effects can be engineered as a function of ***t*** and ***n*** as shown in Fig. 3c,d. Calculated angular scattering of 100 nm Au nanoparticles with 40 nm dielectric coating and refractive index of 3 demonstrates >3 × light scattering intensities when compared with that of the unshelled one.

The light scattering of a coated sphere was first solved by Aden and Kerker in 1951 and followed by some improved algorithms^42, 43^. In general, the scattering efficiency, *k*_*sca*_, is determined by Eq. (1) in our analysis:

1 $${k}_{sca}=\frac{2}{{\alpha }_{1}^{2}}\sum _{n=1}^{\infty }(2n+1)({|{a}_{n}|}^{2}+{|{b}_{n}|}^{2})$$

where *α*_1_ is size parameters, *a*_*n*_ and *b*_*n*_ are Mie scattering coefficients^43^. As the refractive index of surrounding material increases, there is a red shift of the surface plasmon resonance. On the other hand, the absorption cross section will decreases^29^. Therefore, it is difficult to judge the positive effect of core-shell nanoparticles. The scattering-to-absorption ratio is a criterion to evaluate the relationship between σ_scat_ and σ_abs_. We present the calculated relationships between the ***S*** and ***t***, ***S*** and ***n*** in Fig. 3e,f, respectively. When the coating thickness exceeds 25 nm, ***S*** increases dramatically and the maximum enhancement occurs in 500–820 nm, where the solar spectrum has maximum irradiative intensity. This can be attributed to the size increase of the nanoparticle. The medium enhancement (***S*** > 10) locates in the near-infrared region with thin coating (<20 nm), meaning that the beneficial field enhancement exceeds the punitive absorption significantly in the near-infrared region where Si absorbs weakly and a very small proportion of the scattered energy is wasted as heat rather than radiated. In Fig. 3f, the evident ***S*** enhancement still occurs at near-infrared region. However, as the ***n*** increases, excitation of higher order resonances appears, as illustrated in the top left corner of Fig. 3f (e.g. quadruple moments).

### Core-shell nanoparticles located in front of thin film solar cells

**Figure 4:**
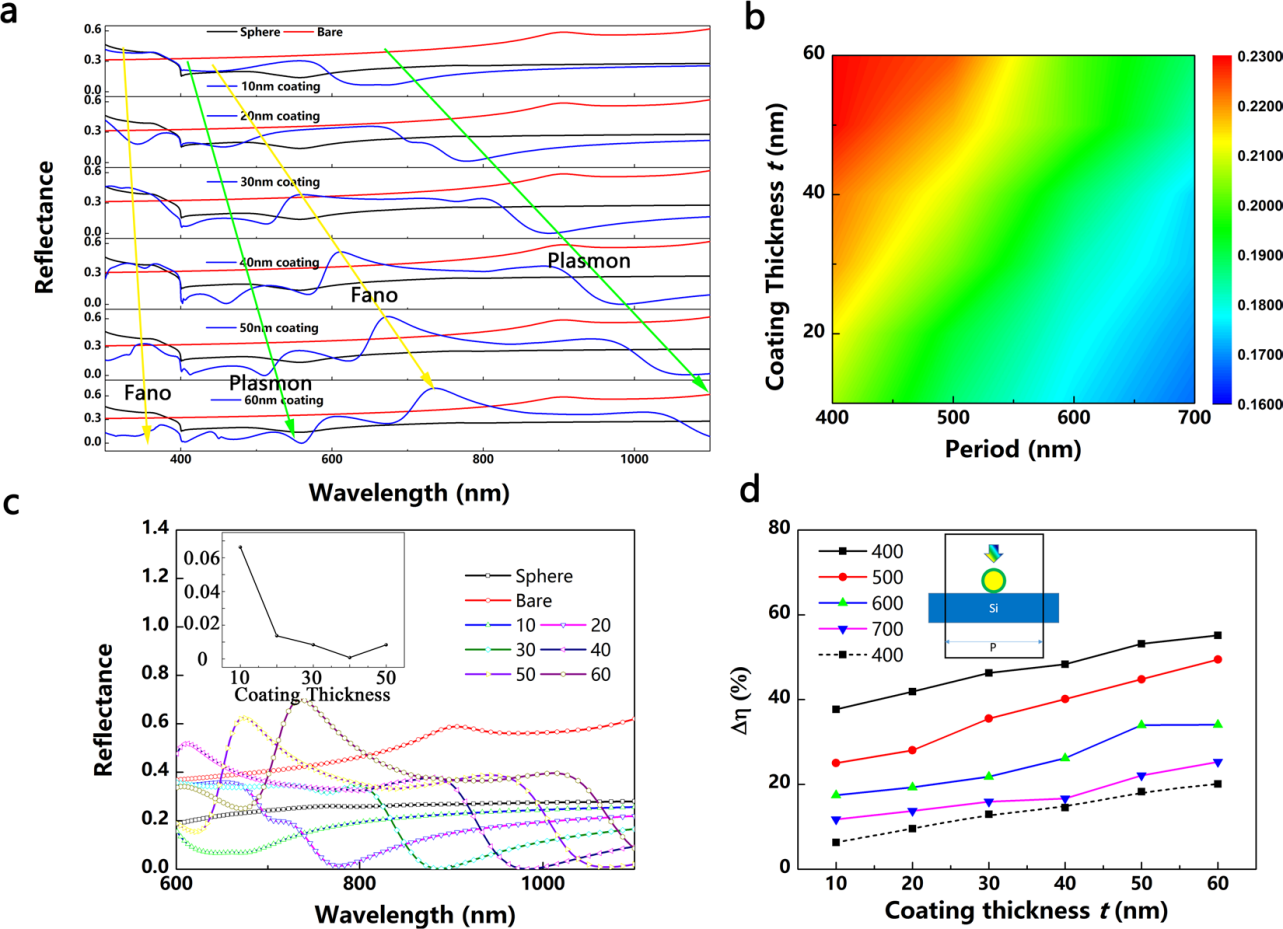
Optical properties and ultimate efficiencies of core-shell nanoparticles with different coating thickness ***t*** (Au core/dielectric shell) placed on the front side of a 2 μm thick Si substrate with different period (***P***). The inner Au core is 120 nm and ***n*** is set as 3.4. (**a**) Reflectance spectra of a flat thick Si (2 μm, bare), a 120 nm Au sphere and core-shell nanoparticles on the 2 μm thick Si as a function of wavelength, the ***P*** is set as 400 nm. (**b**) Ultimate efficiency of core-shell nanoparticles on the 2 μm thick Si. (**c**) Magnified reflectance spectra of a 2 μm thick Si, a 120 nm Au sphere and core-shell nanoparticles on the 2 μm thick Si at long wavelength region; the inset illustrates the minimum reflectance of the 2 μm thick Si with core-shell nanoparticles locating on the surface. (**d**) Differential efficiency ∆η between the core-shell nanoparticles on a 2 μm flat Si and a 2 μm flat Si without nanoparticles. The dashed line is the differential value of efficiency between the core-shell nanoparticles on a 2 μm thick Si and a 120 nm Au sphere on a 2 μm thick Si and the ***P*** = 400 nm; the inset is the schematic in simulation.

According to the abovementioned discussion of core-shell nanoparticle, we modelled the core-shell nanoparticle on a 2 μm thick Si substrate without any front or back anti-reflective coating to study the effect of their optical properties on the front/back surface. Figure 4 demonstrates their optical properties and ultimate efficiency without taking electrical losses into account. The reflectance curve shows a succession of peaks and dips which are indicated by the arrows (green plasmon/yellow Fano), as shown in Fig. 4a. The reflection peaks can be attributed to the Fano resonance which is inherent to plasmonic nanostructures and metamaterials^44^. When the interference wavelength is lower than the plasmon resonance, a phase difference π is generated compared to the incident field, and thus give rise to destructive interference, lowering the electric field amplitude in the silicon. On the other hand, the dips can be ascribed to plasmon resonance. For wavelengths, longer than the resonance wavelength, the light is scattered preferentially towards the high-index Si medium, keeping in phase with the incident field. It is worth noting that, as the coating thickness increases, the reflectance peak increases, indicating that the Fano resonance becomes evident with increasing dielectric coating thickness^31^. As discussed in relation to Fig. 2, although the scattering intensity of the unshelled structure is stronger than that of the core-shell one, it contributes to the useless backward scattering which is detrimental to the absorption. Fortunately, the further suppression of backward reflectance at longer wavelengths can compensate these loss mechanisms, as the ultimate efficiency shown in Fig. 4b. Figure 4c plots the magnified reflectance of the nanoparticles on the Si substrate at long wavelengths. The bare 2 μm thick Si demonstrates a relatively high reflectance at long wavelengths while the presence of the unshelled Au nanoparticles suppresses the reflectance by utilizing the plasmonic effect. Compared with the flat silicon or the silicon with front-located bare Au nanoparticles, the dielectric-coated nanoparticles can minimize the reflectance to zero at near-infrared region preserved over a broadband spectral range, and thus lead to an enhanced ultimate efficiency, as illustrated in Fig. 4d.

**Figure 5:**
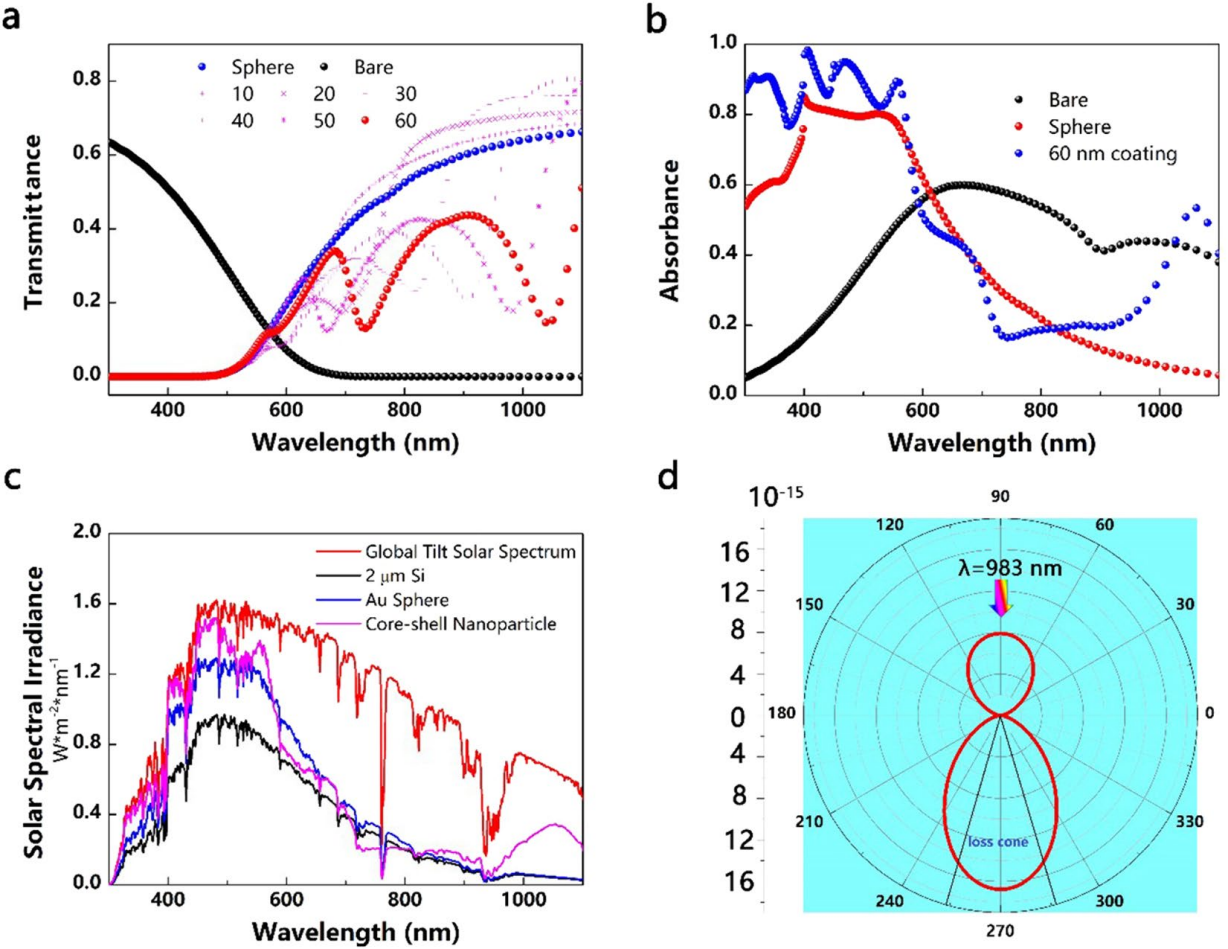
(**a**) Transmittance spectra of a 2 μm thick Si, a 120 nm Au sphere and core-shell nanoparticles with different coating thickness ***t*** on the 2 μm thick Si substrates. (**b**) Comparison of absorption spectra among a 2 μm thick Si, a 120 nm Au sphere and core-shell nanoparticles with a 60 nm coating on the 2 μm thick Si substrates and the ***P*** is set as 400 nm. (**c**) Global tilt solar spectrum, together with graphs that illustrate the solar energy absorbed in a 2 μm thick Si film (assuming single-pass absorption and no reflection coating), a 2 μm thick Si film with a 120 nm Au sphere (***P*** = 400 nm) and a 2 μm thick Si film with a core-shell nanoparticle (***t*** = 60 nm, ***P*** = 400 nm). (**d**) Angular scattering of a core-shell nanoparticle on a 2 μm thick Si film under 983 nm illumination (***t*** = 40 nm, ***P*** = 400 nm), indicating the suppressed backward scattering and the favoured forward scattering.

As the particle size increases, the resonant mode (i.e. dipole, quadrupole, and hexapole) redshifts with an increased scattering into underneath substrate and decreased absorption in metal nanoparticle^45^. The positive effect of the improved light trapping can compensate the negative effect of the loss mechanism (parasitic absorption and destructive interference of Fano resonance), giving a positive total gain from the plasmonic effects integrated over the overall solar spectrum, as demonstrated in Fig. 5a–c). The light absorption in the UV-vis region is notably boosted. For a flat 2 μm Si, light leaks out of the layer after a single passage, but the presence of plasmonic nanoparticles aids the path length enhancement. The 2 μm thick Si without plasmonic enhancer demonstrates high reflectance and low transmittance in the near infrared region, but the 2 μm thick Si with Au sphere or core-shell nanoparticle shows suppressed reflectance and increased transmittance, as shown in Figs 4c and 5a. The increased transmittance indicates the enhanced path length enhancement and a higher possibility to be trapped by the substrate. As discussed above, the 60 nm coating core-shell nanoparticle boosts the cell performance with maximum ultimate efficiency. The enhanced absorption mainly originates from increased absorption in the short wavelength and long wavelength above 1000 nm, as plotted in Fig. 5b. However, there is a dramatic improvement in short wavelength region compared to minor improvement in long wavelength region. The reason can be ascribed to the ultra-thin Si film and its high absorption in the short wavelength region. The short wavelength light is absorbed at the surface and the near-field enhancement contributes significantly because the particle and the Si film are adjacent. On the other hand, the far-field scattering beneficiation dominates at long wavelength far away from the surface. Although it suffers from loss mechanism because of destructive interference of Fano resonance, the 2 μm Si with the core-shell nanoparticles still demonstrates broadband enhancement, as solar spectra absorption demonstrated in Fig. 5c. To provide an insight into the suppressed backward reflectance, the angular scattering intensity at 983 nm for the 40 nm coating nanoparticle on a 2 μm Si is plotted. The forward scattering exhibits a more evident scattering into the silicon substrate than that of the backward scattering to air. In the 40 nm coating case, the fraction of light scattered into the substrate is 99.9% while the bare case is 43.8% and the Au sphere is 72%, respectively. The electric and magnetic dipoles interfere destructively in the backward direction and constructively in the forward direction^32^. The loss cone for a Si/Air interface is defined by the critical angle, *θ*_*c*_ = arcsin(1/n_si_), as illustrated in Fig. 5d. As shown in figure, 91% of the forward scattered light is trapped at 983 nm. It is worth noting that the enhanced forward scattering with zero backward scattering can be further enhanced by using Ag core and increasing coating dielectric thickness^32^.

### Rear-located core-shell nanoparticles on thin film solar cells

**Figure 6:**
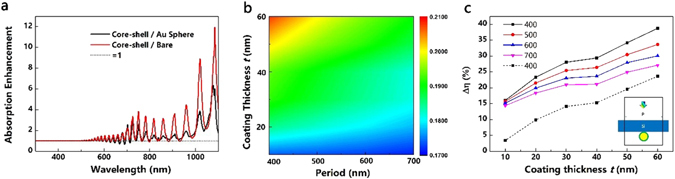
Optical properties and ultimate efficiencies of core-shell nanoparticles with different ***t*** placed on the rear side of the 2 μm thick Si substrates with different ***P***. The inner Au core is 120 nm and the ***n*** is set as 3.4. (**a**) Absorption enhancement factor of core-shell nanoparticle when compared with the 2 μm thick Si with 120 nm Au sphere and a flat cell (***P*** = 400 nm, ***t*** = 60 nm). (**b**) Ultimate efficiency of core-shell nanoparticles on the 2 μm thick Si. (**c**) Differential efficiency ∆η between the core-shell nanoparticles on a 2 μm flat Si and a 2 μm flat Si without nanoparticles. The dashed line is the differential value between the core-shell nanoparticles on a 2 μm thick Si and a 120 nm Au sphere on a 2 μm thick Si and the ***P*** is set as 400 nm; the inset is the schematic in simulation.

**Figure 7:**
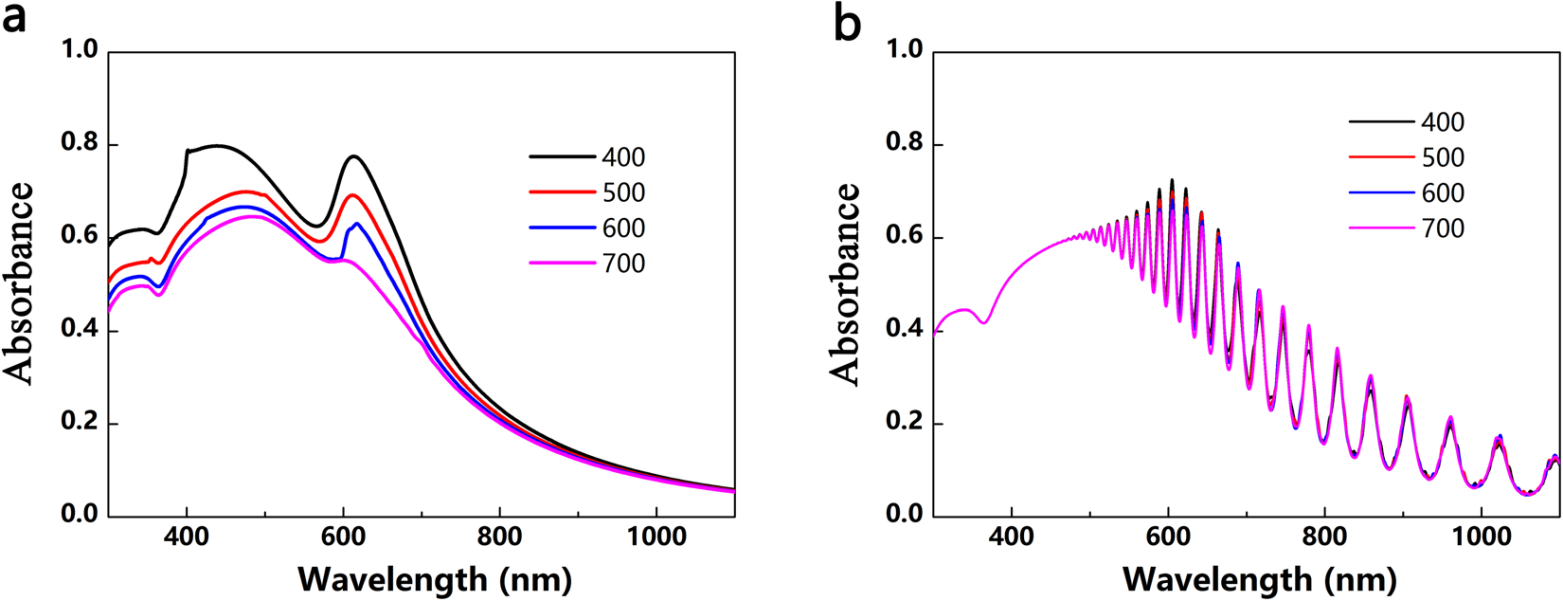
Absorption spectra of core-shell nanoparticles on the 2 μm thick Si substrates with fixed ***t*** = 10 nm and different ***P***. (**a**) Front. (**b**) Rear.

Placing the nanoparticles on the front surface of solar cells introduces destructive interference with weakened absorption at shorter wavelengths than the plasmon resonance, while placing them on the rear of solar cells may help to avoid such undesirable effects^46^. Figure 6a depicts the absorption enhancement factor for core-shell nanoparticles compared with Au sphere on solar cells and the flat solar cells. The oscillations in the longer wavelengths can be attributed to Fabry-Perot resonances and the waveguide modes in an ultra-thin wafer. While the front-located nanoparticle augment light harvesting predominately occurs in the shorter wavelengths, the narrow spectral features (R, T and A) enhancement appears at longer wavelengths in the rear-located architecture. This phenomenon can be explained by higher absorption of Si at shorter wavelengths, but its poor absorption in longer wavelength. However, the ultimate efficiencies in Fig. 6b contradict with previous work that the rear side of the cell is the most effective location for plasmonic scatters^46, 47^. Under the same illumination direction, the core-shell nanoparticles can support simultaneously both electric and magnetic resonances with vanishing backward scattering and enhanced forward scattering, indicating more leaked light due to forward scattering when they are located on the rear surface. Figure 6c demonstrates ∆η between the core-shell nanoparticles on the 2 μm thick Si and reference cell. The ultimate efficiencies increase with the mounting ***t*** and ***P***, as the trend shown in Fig. 4d. However, the ultimate efficiencies for core-shell nanoparticles with thin coating are slightly influenced by the ***P*** changes. Per the absorption spectra of core-shell nanoparticles with fixed coatings 10 nm at different ***P***, they remain unchanged, as shown in Fig. 7b. In the front case, the Si film absorbs the short wave at its surface with dominating near-filed enhancement contribution from core-shell nanoparticles, as shown in Fig. 7a ^20^. However, the core-shell nanoparticles are too small to interact with adjacent ones no matter how the ***P*** changes when the light penetrates to the bottom where far-field scattering dominates. When their sizes are small, they contribute absorption enhancement independently via near-field enhancement or light scattering.

**Figure 8:**
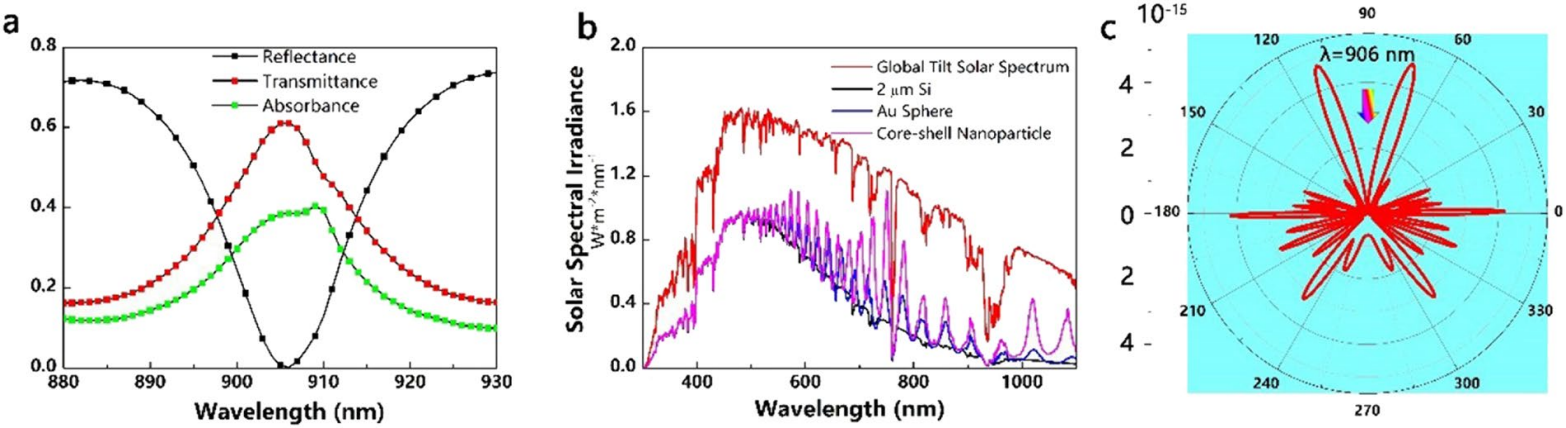
(**a**) Characteristic line shape of a rear-located core-shell nanoparticle on a 2 μm thick Si (***t*** = 60 nm, ***P*** = 400 nm). (**b**) Global tilt solar spectrum, together with graphs that illustrate the solar energy absorbed in a 2 μm thick Si film (assuming single-pass absorption and no reflection coating), a 2 μm thick Si film with a 120 nm Au sphere (***P*** = 400 nm) located on the rear surface and a 2 μm thick Si film with a core-shell nanoparticle (***t*** = 60 nm, ***P*** = 400 nm). (**c**﻿) Angular scattering of a core-shell nanoparticle on a 2 μm thick Si film under 906 nm illumination (***t*** = 60 nm, ***P*** = 400 nm).

A reflectance dip is accompanied by a transmittance peak in near-infrared region, leading to an absorption enhancement peak, as shown in Fig. 8a,b. Referring to Fig. 6a, the absorption spectra exhibit interference fringes at the long wavelength, and contribute to the ultimate efficiency enhancement. The angular distribution of scattered light for core-shell nanoparticle is taken at minimum reflection. An evident out-coupling of core-shell nanoparticle on the rear side is stronger than that of the front side, as demonstrate in Figs 5d and 8c. This again justifies the fact that the ultimate efficiency of solar cells with front-located nanoparticle outperforms that of rear-located ones. While rear nanoparticles have a larger light incoupling efficiency than the front nanoparticles in bare metal nanoparticle-assisted solar cells, the core-shell nanoparticle-assisted solar cells demonstrate contrary phenomenon. Additionally, the rear-located one has limited scattering angles. For bare nanoparticles, small size particles are able to scatter the incident light into a larger angle range but lower scattering intensities while large size particles have stronger scattering intensities but limited scattering angles^25^. However, the large size core-shell nanoparticles with thick coating thicknesses have strong scattering intensities while maintaining comparable scattering intensities versus angle distribution when located at the front side. The results suggest that the large size stratified nanoparticles can scatter the incident light into a larger angle range providing a higher possibility for light trapping Si thin film solar cells.

## Discussion

We simulated the influence of core (metal)-shell (dielectric) nanoparticles on the optical absorption enhancement in thin film Si solar cells. The optical scattering cross section of core-shell nanoparticle depends on the surrounding dielectric environment and coating thickness that exerts influence on the solar cell applications. Core-shell nanoparticles located on the rear and front sides of a 2 μm Si thin film enable broadband solar light capture with distinct absorption enhancement; the front-located nanoparticle enhances absorption in the short wavelength region while the rear-located nanoparticle boost absorption at the long wavelength. Although the solar cells with front-located nanoparticles are subjected to destructive interference at shorter wavelengths than the plasmon resonance, they still outperform the solar cell with rear-located ones. This phenomenon is contrary to the reported simulation and results based on bare metallic plasmonic enhancers because of the specific property of core-shell nanoparticles: enhanced forward scattering with vanishing backward scattering if properly engineered. This work provides a new insight for devising plasmonic enhancers in thin film solar cells with favorable forward scattering, suppression of reflection and minimum heat loss.

## Methods

All the simulations are carried out using the finite difference time domain (FDTD) method with FDTD solutions (Lumerical) and online multilayer nanoparticle toolkit^48^. The refractive index of materials in simulation are modelled using fitted optical data for Au and Si^49^. We decrease the mesh size until the results are convergent. Absorption cross-section (σ_abs_) and scattering cross-section (σ_scat_) are calculated following the Mie scattering theory. Two built-in analysis groups are surrounded by the particle and a total-field scattered-field (TFSF) source is injected. One group is for the total field and another is for the scattered field. The background refractive index is set to 1. The perfectly matched layer (PML) boundary condition and the periodic boundary condition are adopted for simulation. To evaluate the performance of solar cells, we calculated the ultimate efficiency, *η*, which is defined by Eq. (2)

2 $$\eta =\frac{{\int }_{0}^{{\lambda }_{g}}{P}_{AM1.5}(\lambda )A(\lambda )\frac{\lambda }{{\lambda }_{g}}d\lambda }{{\int }_{0}^{\infty }{P}_{AM1.5}(\lambda )d\lambda }$$

where *λ* is the wavelength, *A(λ)* is wavelength-dependent absorption which is characterized by *A(λ)* = *1−R(λ)−T(λ)*, *P*_*AM1*.*5*_ is the solar spectral power density, *λ*_*g*_ is the wavelength corresponding to the band gap of Si.
